# The Identification and Experimental Validation of Monocyte/Macrophage-Related Inflammatory Hub Genes in Ventilator-Induced Lung Injury

**DOI:** 10.7759/cureus.112627

**Published:** 2026-07-13

**Authors:** Wenhao Liu, Yaming Liu, Zhiyong Zeng

**Affiliations:** 1 Department of Thoracic Surgery, 900th Hospital of PLA Joint Logistic Support Force, Fuzhou, CHN; 2 Department of Thoracic Surgery, Fuzong Clinical Medical College of Fujian Medical University, Fuzhou, CHN

**Keywords:** ccr1, immune infiltration, machine learning, monocyte/macrophage, ventilator-induced lung injury

## Abstract

Objective

This study aimed to identify monocyte/macrophage-related hub genes in ventilator-induced lung injury (VILI) by integrating transcriptomic analysis, immune infiltration analysis, weighted gene co-expression network analysis (WGCNA), protein-protein interaction (PPI) network analysis, and machine learning approaches, followed by experimental validation using a VILI animal model.

Methods

Seven mouse lung transcriptomic datasets related to VILI were obtained from the Gene Expression Omnibus (GEO) database and integrated after normalization and batch effect correction. Differential expression analysis was performed to identify VILI-associated genes. Immune infiltration analysis and WGCNA were applied to characterize changes in the pulmonary immune microenvironment and identify modules correlated with monocyte/macrophage infiltration. Key module genes were intersected with VILI-related differentially expressed genes (DEGs) and subsequently subjected to Gene Ontology (GO), Kyoto Encyclopedia of Genes and Genomes (KEGG), and PPI analyses. Based on monocyte/macrophage infiltration levels, samples were stratified, and candidate genes were screened using least absolute shrinkage and selection operator (LASSO) regression, support vector machine-recursive feature elimination (SVM-RFE), and the random forest algorithm. Receiver operating characteristic (ROC) curve analysis was then performed to evaluate the discriminatory performance of the selected genes. Finally, experimental validation was conducted using histological analysis, bronchoalveolar lavage fluid cytokine assays, quantitative real-time polymerase chain reaction (qPCR), and immunofluorescence staining.

Results

Lung tissues from VILI model mice showed marked remodeling of the immune microenvironment, characterized by increased proportions of monocytes/macrophages and neutrophils, along with reduced CD4 T cell- and innate lymphoid cell-related populations. The MEblack module was positively correlated with monocyte/macrophage infiltration (r = 0.64) and was selected as the key module. Intersecting MEblack genes with VILI-related differentially expressed genes identified 99 candidate genes mainly enriched in chemotaxis, inflammatory response regulation, leukocyte migration, and IL-17, TNF, and NF-κB signaling pathways. Stratification by monocyte/macrophage abundance further identified 82 common differentially expressed genes with similar enrichment patterns. Three machine-learning algorithms jointly prioritized Ccr1, Mmp9, and Il36g as core feature genes, all of which were upregulated in VILI. ROC analysis showed the strongest discriminatory performance for Ccr1 (area under the curve (AUC) = 0.987), followed by Il36g (AUC = 0.891) and Mmp9 (AUC = 0.883). Experimental validation confirmed inflammatory infiltration, structural lung injury, elevated IL-1β, IL-6, and TNF-α levels, increased expression of the three hub genes, and enhanced CCR1-positive signals in alveolar septal and perivascular regions.

Conclusions

VILI is associated with monocyte/macrophage infiltration and activation of inflammatory chemotaxis-related pathways. Ccr1, Mmp9, and Il36g may serve as monocyte/macrophage-related inflammatory feature genes, with Ccr1 showing particular promise as a candidate biomarker of pulmonary inflammatory activation in VILI.

## Introduction

Mechanical ventilation is an essential life-support strategy for critically ill patients. However, excessive or prolonged mechanical stretch and strain may aggravate lung injury and contribute to ventilator-induced lung injury (VILI). The development of VILI involves several interrelated mechanisms with distinct anatomical correlates. Volutrauma primarily results from overdistension of alveoli and lung parenchyma, whereas barotrauma is associated with pressure-related alveolar rupture and extra-alveolar air leakage. Atelectrauma reflects the repetitive opening and collapse of unstable alveoli and small airways, particularly in dependent lung regions. In contrast, biotrauma is characterized by the release of inflammatory mediators and the activation of epithelial, endothelial, and immune cells within the alveolar-capillary unit [1,2]. Together, these processes lead to alveolar epithelial damage, endothelial barrier dysfunction, pulmonary edema, and persistent local inflammation. Mechanical ventilation may also exert systemic effects through cytokine release and the mobilization of inflammatory cells [3,4].

The alveolar and interstitial compartments of the lung contain abundant innate immune cells. Among them, monocytes and macrophages are central regulators of pulmonary immune homeostasis. Alveolar macrophages contribute to tissue surveillance by clearing apoptotic cells and damage-associated molecular patterns, and they also participate in inflammatory resolution and repair. In response to mechanical stretch or tissue injury, however, monocytes and macrophages may shift toward a pro-inflammatory state. Activated monocyte/macrophage produce IL-1β, IL-6, TNF-α, chemokines, and matrix-degrading enzymes, thereby promoting inflammatory cell infiltration and disruption of the alveolar-capillary barrier [5,6]. Previous studies of VILI have also implicated recruited pulmonary monocytes and neutrophils in inflammatory amplification and subsequent tissue repair [6-8].

Transcriptomic analyses have advanced our understanding of molecular alterations associated with VILI. Differential expression analysis combined with weighted gene co-expression network analysis (WGCNA) has been applied to screen gene modules and potential biomarkers associated with VILI [9]. However, monocyte/macrophage-related genes linking transcriptional alterations, immune infiltration, and diagnostic relevance in VILI remain insufficiently characterized. The objective of this study was to identify and validate monocyte/macrophage-related inflammatory hub genes involved in the pathogenesis of VILI using integrated transcriptomic analyses and experimental validation. We hypothesized that VILI is associated with marked remodeling of the lung immune microenvironment, particularly involving monocyte/macrophage-related inflammatory responses, and that specific immune-related hub genes may serve as potential molecular markers of VILI. To test this hypothesis, we integrated differential expression analysis, WGCNA, immune infiltration analysis, machine-learning-based feature selection, and validation in an experimental VILI mouse model. This study may provide new insights into monocyte/macrophage-associated inflammatory mechanisms in VILI and help identify candidate biomarkers with potential diagnostic relevance for future validation.

## Materials and methods

Overall study design

This study was conducted in two sequential phases: a bioinformatics identification phase and an experimental validation phase. In the identification phase, publicly available VILI-related GEO transcriptomic datasets were analyzed to identify monocyte/macrophage-related inflammatory hub genes through differential expression analysis, WGCNA, immune infiltration analysis, protein-protein interaction (PPI) network analysis, machine-learning algorithms, and receiver operating characteristic (ROC) curve analysis. In the validation phase, the selected candidate genes were further validated in an experimental VILI mouse model using histological assessment, inflammatory cytokine detection, quantitative real-time polymerase chain reaction (qPCR), and immunofluorescence staining.

Identification phase: bioinformatics screening of candidate hub genes

Data Acquisition and Differential Expression Analysis

The GSE9314, GSE9368, GSE11434, GSE11662, GSE18341, GSE85269, and GSE86229 datasets were retrieved from the Gene Expression Omnibus (GEO) database through the GEOquery package [10]. Detailed information on the included datasets is provided in Table 1. Datasets were included if they contained lung samples from control mice that were spontaneously breathing or not mechanically ventilated and from experimental mice subjected to injurious mechanical ventilation. The included VILI models mainly involved high-tidal-volume or pressure-controlled ventilation protocols. Detailed ventilation settings, including tidal volume or driving pressure, respiratory rate, positive end-expiratory pressure, and ventilation duration when available, are summarized in Table 1. All microarray probes were converted to their corresponding gene symbols and normalized using R. When several probes corresponded to a single gene, their expression values were averaged to generate one representative value for downstream analysis. To minimize systematic differences across microarray platforms and batches, batch-effect correction was performed using the Surrogate Variable Analysis package [11]. Differential expression analysis was conducted with the limma package using linear model fitting and empirical Bayes moderation [12,13]. Differentially expressed genes were screened using the criteria of |logFC| > 1 and adjusted p < 0.05. The overall distribution and expression patterns of these genes were displayed using volcano plots and heatmaps.

**Table 1:** Characteristics of included GEO datasets GEO: Gene Expression Omnibus; GSE: GEO Series accession number; GPL: GEO Platform accession number; VILI: ventilator-induced lung injury

Accession	Platform	Sample	Control group	VILI group	Ventilation settings
GSE9314	GPL1261	Lung tissue	4	4	MV, VT 30 mL/kg, 4 h
GSE9368	GPL1261	Lung tissue	3	3	MV, VT 30 mL/kg, 4 h
GSE11434	GPL1261	Lung tissue	5	5	MV, driving pressure 20 cmH₂O, 3 h
GSE11662	GPL1261	Lung tissue	3	3	MV, VT 30 mL/kg, 4 h
GSE18341	GPL1261	Lung tissue	4	4	MV, VT 15 mL/kg, 2 h
GSE85269	GPL16750	Lung tissue	3	3	MV, driving pressure 15 cmH₂O, 2.5 h
GSE86229	GPL6246	Lung tissue	5	10	MV, VT 35 mL/kg, 3 h

Immune Infiltration Analysis

Immune cell infiltration was estimated using the batch-corrected bulk transcriptomic expression matrix as the mixture matrix and a mouse immune cell-specific signature matrix as the reference. The CIBERSORT deconvolution algorithm was used to estimate the relative abundance of immune cell subsets from bulk tissue expression profiles [14]. In addition, mouse immune cell composition inference was performed as described by Chen et al. [15]. To facilitate biological interpretation of the deconvolution results, the inferred immune cell subsets were grouped into nine major immune cell categories according to lineage and functional characteristics, and the representative grouping strategy used in previous ImmuCC-based deconvolution studies [16]. These categories included B cells, CD4/ILC cells, CD8 cells, eosinophils, mast cells, monocyte/macrophage, neutrophils, NK cells, and plasma cells. The relative abundance of each immune cell category was compared between the VILI and control groups. Boxplots and heatmaps were generated to visualize differences in immune cell infiltration patterns between groups.

Identification of Monocyte/Macrophage-Related Modules by WGCNA

WGCNA was used to construct a gene co-expression network [17]. Samples were first clustered according to expression profile similarity, and outlier samples were excluded. To construct a scale-free co-expression network, an optimal soft-thresholding power was first determined. The adjacency relationships were subsequently transformed into a topological overlap matrix, and modules of co-expressed genes were defined using the dynamic tree cut method. Correlations between module eigengenes and immune cell proportions were calculated to identify the module most strongly associated with monocyte/macrophage infiltration. According to the module-trait correlation results, the MEblack module, which showed a significant positive correlation with monocyte/macrophage infiltration, was selected for subsequent analysis.

Candidate Gene Screening and Functional Enrichment Analysis

MEblack module genes were intersected with VILI-related differentially expressed gene to obtain monocyte/macrophage-related candidate genes in VILI. Functional enrichment analysis was performed using clusterProfiler, including Gene Ontology Biological Process (GO-BP) and Kyoto Encyclopedia of Genes and Genomes (KEGG) pathway analyses, to characterize the potential biological functions of these genes [18]. A PPI network was constructed using the STRING database, and highly connected core nodes were screened [19].

Differential Expression Analysis Based on Monocyte/Macrophage Abundance

Samples were separated into high- and low-infiltration subgroups using the median monocyte/macrophage abundance as the cutoff, allowing further investigation of genes associated with monocyte/macrophage infiltration in VILI. Differential expression analysis was performed between the two groups. The resulting gene set was intersected with VILI-related differentially expressed gene to identify genes associated with both VILI status and high monocyte/macrophage infiltration. Heatmap visualization and functional enrichment analysis were then performed.

Machine Learning-Based Feature Gene Screening

Hub genes were further screened using an integrated machine-learning strategy based on least absolute shrinkage and selection operator (LASSO) regression, support vector machine-recursive feature elimination (SVM-RFE), and random forest analysis. LASSO regression was performed using the glmnet package with alpha set to 1, and the optimal penalty parameter was selected by cross-validation [20]. SVM-RFE was used to recursively remove redundant features and identify the optimal feature subset. Random forest analysis was performed to rank candidate genes according to variable importance and out-of-bag error, based on the algorithm proposed by Breiman [21]. Genes shared by the three algorithms were defined as VILI core feature genes. Because no independent external cohort was available for repeated feature stability testing, the machine-learning results were considered exploratory and require further validation.

ROC Curve Analysis

The expression profiles of the core feature genes were extracted for the VILI and control groups. Diagnostic performance was assessed using ROC curve analysis with the pROC package, and the corresponding area under the curve (AUC) values were calculated for each gene [22].

Software and Package Versions

All bioinformatics analyses were performed using R software (version 4.5.0). Differential expression analysis was conducted using the limma package (version 3.64.3). Heatmaps were generated using the pheatmap package (version 1.0.13). Weighted gene co-expression network analysis was performed using the WGCNA package (version 1.74). LASSO regression was performed using the glmnet package (version 4.1.10). Support vector machine analysis was conducted using the e1071 package (version 1.7.17), and random forest analysis was performed using the randomForest package (version 4.7.1.2). ROC curve analysis was performed using the pROC package (version 1.19.0.1). Data visualization was performed using ggplot2 (version 4.0.2) [23].

Validation phase: experimental validation in a VILI animal model

Animal Model and Ethics Statement

Male C57BL/6J mice aged six to eight weeks were provided by the Experimental Animal Center of Fujian Medical University. The animal license number was SYXK (Min) 2018-0006. The experimental protocol complied with the ethical requirements for laboratory animal welfare and was approved by the Laboratory Animal Welfare and Ethics Committee of the 900th Hospital of PLA Joint Logistic Support Force (Approval No. 2024-08). All animal experiments were carried out in accordance with relevant local regulations and the guidelines of the institutional animal ethics committee.

Mice were randomly divided into two groups, the control group and the VILI group, with five animals assigned to each group. Before the experimental procedures, anesthesia was induced by intraperitoneal administration of 1% pentobarbital sodium at a dose of 50 mg/kg. After adequate anesthesia, mice were fixed on an operating table and underwent tracheal intubation. Control mice underwent tracheal intubation and remained under anesthesia without mechanical ventilation. Mice in the VILI group were connected to a small-animal ventilator and subjected to high-tidal-volume mechanical ventilation at 30 mL/kg for four hours. Body temperature was maintained during the experiment, and respiratory status and general vital signs were monitored. After four hours, blood was collected by cardiac puncture under anesthesia, and mice were euthanized by overdose anesthesia. Lung tissues were harvested after cessation of breathing, and bronchoalveolar lavage fluid was collected. Lung tissues were used for hematoxylin and eosin (H&E) staining, quantitative polymerase chain reaction, and immunofluorescence staining. Bronchoalveolar lavage fluid was used to measure IL-1β, IL-6, and TNF-α levels.

H&E Staining

For histological analysis, lung tissues were preserved in 4% paraformaldehyde, processed for paraffin embedding, and sectioned. The prepared sections were then deparaffinized, rehydrated, and stained with H&E using a staining kit from Beyotime Biotechnology (Shanghai, China) according to the manufacturer’s instructions. Alveolar structure, alveolar septal thickness, inflammatory cell infiltration, and tissue injury were observed under a light microscope (Olympus, Tokyo, Japan).

Inflammatory Cytokine Detection

Bronchoalveolar lavage fluid was collected, and IL-1β, IL-6, and TNF-α levels were measured using commercial enzyme-linked immunosorbent assay kits (Servicebio, Wuhan, China) according to the manufacturer's instructions to assess local pulmonary inflammation in the VILI model.

RNA Extraction and Quantitative Polymerase Chain Reaction

Total RNA was extracted from lung tissues using TRIzol reagent (Servicebio). RNA concentration and purity were measured using a NanoDrop 2000 spectrophotometer (Thermo Fisher Scientific, Waltham, MA). RNA was reverse transcribed into complementary DNA using a reverse transcription kit (Vazyme Biotech, Nanjing, China) according to the manufacturer's instructions. Real-time quantitative PCR was performed using SYBR Green qPCR Master Mix (Vazyme Biotech) on a real-time PCR detection system to detect the messenger RNA expression levels of Ccr1, Mmp9, and Il36g. Gapdh was used as the internal reference gene. Primer information is summarized in Table 2. The relative mRNA expression levels were quantified with the comparative Ct method, and fold changes were calculated using the 2−ΔΔCt formula described by Livak and Schmittgen [24].

**Table 2:** Specific primer sequences used in quantitative polymerase chain reaction Ccr1: C-C motif chemokine receptor 1; Mmp9: matrix metalloproteinase 9; Il36g: interleukin 36 gamma; Gapdh: glyceraldehyde-3-phosphate dehydrogenase

Gene	Forward primer (5'-3')	Reverse primer (5'-3')
Ccr1	GCCAAAAGACTGCTGTAAGAGCC	GCTTTGAAGCCTCCTATGCTGC
Mmp9	CTTCTGGCGTGTGAGTTTCCA	ACTGCACGGTTGAAGCAAAGA
Il36g	TTGACTTGGACCAGCAGGTGTG	GGGTACTTGCATGGGAGGATAG
Gapdh	CATCACTGCCACCCAGAAGACTG	ATGCCAGTGAGCTTCCCGTTCAG

Immunofluorescence staining

After deparaffinization, hydration, antigen retrieval, and blocking, lung tissue sections were incubated overnight at 4°C with a rabbit anti-CCR1 primary antibody (ABclonal Technology, Wuhan, China; catalog number: A18341; dilution: 1:100) and a rat anti-mouse CD31 primary antibody (Thermo Fisher Scientific; catalog number: 14-0311-82; dilution: 1:100). CCR1 was detected using a YSFluor 488-conjugated goat anti-rabbit IgG (H+L) secondary antibody (Yeasen Biotechnology, Shanghai, China; catalog number: 33106ES60; dilution: 1:200), and CD31 was detected using a YSFluor 594-conjugated donkey anti-rat IgG (H+L) secondary antibody (Yeasen Biotechnology; catalog number: 34412ES60; dilution: 1:200). Nuclei were counterstained and sections were mounted using antifade mounting medium with DAPI (Beyotime Biotechnology; catalog number: P0131). CCR1 expression and distribution in lung tissues were observed using an Olympus FV3000 confocal microscope (Olympus), and CD31 staining was used to analyze the spatial relationship between CCR1-positive signals and vascular-associated regions.

Statistical analysis

Data analysis was conducted using R software and GraphPad Prism 11.0.0 (GraphPad Software, Boston, MA). Quantitative results are expressed as the mean ± standard error of the mean (SEM). Differences between two groups were analyzed with Student’s t-test, whereas comparisons involving more than two groups were assessed by one-way analysis of variance (ANOVA). A p-value < 0.05 was considered statistically significant.

## Results

VILI lungs showed marked immune microenvironment remodeling

**Figure 1:**
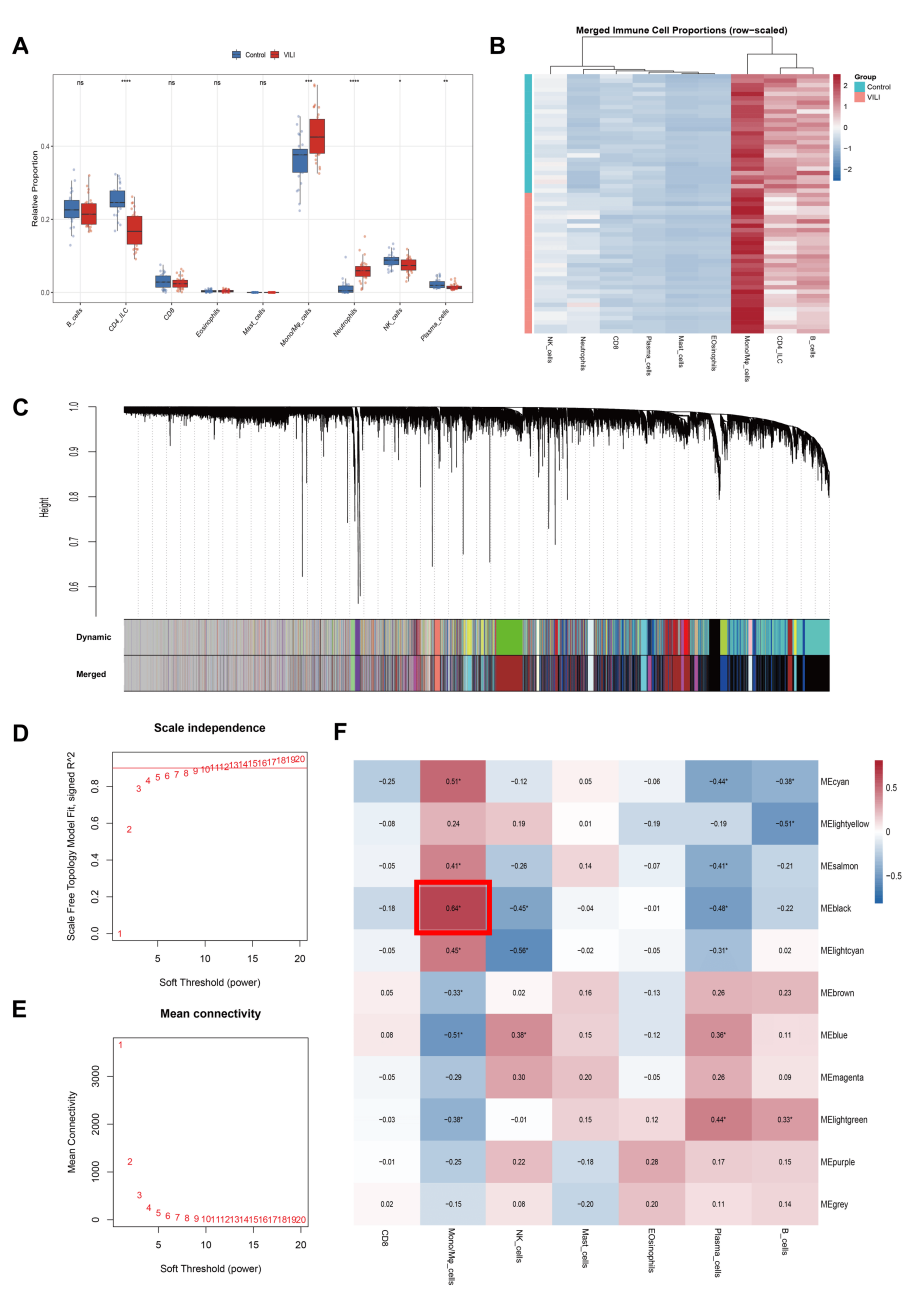
Immune cell infiltration characteristics and WGCNA module screening in VILI lung tissues A: Group-wise comparison of immune cell proportions between VILI and control samples. B: Heatmap visualization of immune cell composition across samples. C: WGCNA gene clustering tree and module assignment. D-E: Soft-thresholding power selection. F: Heatmap showing correlations between modules and immune cell proportions; the MEblack module was significantly positively correlated with monocyte/macrophage VILI: ventilator-induced lung injury; WGCNA: weighted gene co-expression network analysis; MEblack: module eigengene of the black module; CD4/ILC: CD4-positive/innate lymphoid cell-related population; NK: natural killer

Immune infiltration analysis revealed marked alterations in the immune cell landscape of VILI lung tissues compared with control lung tissues (Figure 1A). Compared with the control group, the VILI group showed increased monocyte/macrophage proportions and increased neutrophil proportions, suggesting that mechanical ventilation-induced lung injury was accompanied by marked innate immune cell recruitment. CD4/innate lymphoid cell-related lymphocyte populations were relatively decreased in VILI lungs, indicating a shift in the local pulmonary immune microenvironment from homeostatic defense toward monocyte/macrophage- and myeloid inflammation-dominated responses. Heatmap analysis of immune cell proportions further showed that VILI samples displayed higher monocyte/macrophage-related immune features and were clearly separated from control samples (Figure 1B).

The MEblack module was strongly associated with monocyte/macrophage infiltration

To identify gene modules related to immune alterations in VILI, a WGCNA co-expression network was constructed. Multiple co-expression modules were obtained after dynamic tree cutting and module merging (Figure 1C). Soft-thresholding power selection supported scale-free network construction (Figures 1D, 1E). Correlation analysis between immune cell infiltration and WGCNA modules revealed that the MEblack module was most strongly and positively associated with monocyte/macrophage infiltration, showing a correlation coefficient of 0.64 (Figure 1F). This finding suggested that the MEblack module may contain key genes involved in regulating monocyte/macrophage infiltration or reflecting monocyte/macrophage inflammatory activation. The MEblack module also showed negative correlations with some other immune cell subsets, indicating that it may represent an immune remodeling pattern characterized by enhanced monocyte/macrophage inflammation and relatively reduced adaptive immune components. Therefore, the MEblack module was selected for subsequent analysis.

Intersection of the MEblack module and VILI differentially expressed genes indicated activation of chemotaxis and cytokine signaling

**Figure 2:**
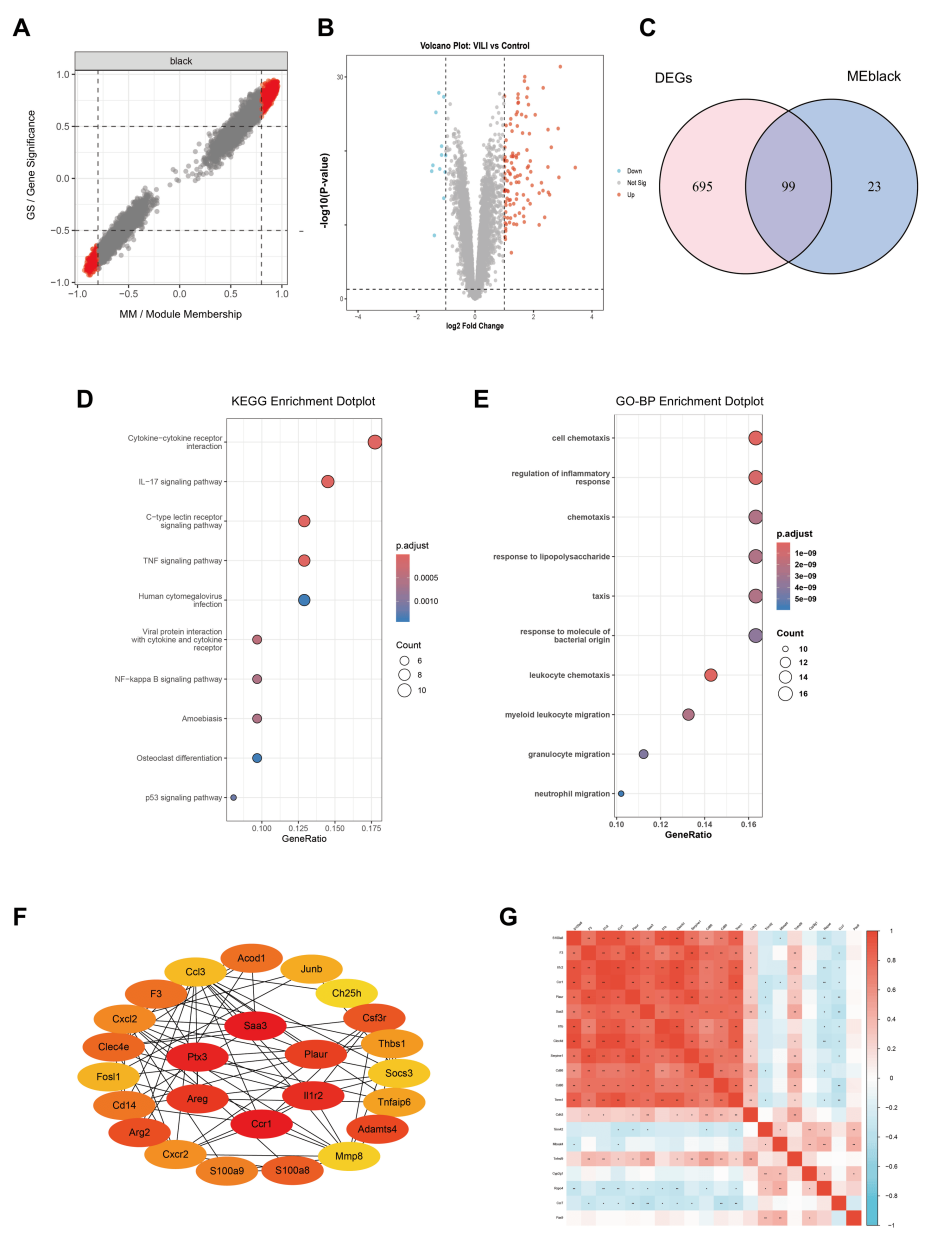
Intersection and functional analysis of MEblack module genes and VILI-related differentially expressed gene A: Correlation between monocyte/macrophage and the black module. B: Volcano plot of VILI-related differentially expressed gene. C: Intersection of MEblack module genes and VILI-related differentially expressed gene. D-E: KEGG and GO enrichment analyses. F: PPI network. G: Correlation heatmap of core candidate genes. In panel A, the x-axis represents module membership, and the y-axis represents gene significance for monocyte/macrophage infiltration. Red points indicate genes meeting the screening criteria of |MM| > 0.8 and |GS| > 0.5, whereas gray points indicate other genes VILI: ventilator-induced lung injury; DEGs: differentially expressed genes; MEblack: black module eigengene; KEGG: Kyoto Encyclopedia of Genes and Genomes; GO: Gene Ontology; PPI: protein-protein interaction; logFC: log fold change

As shown in Figure 2A, module membership was positively associated with gene significance in the MEblack module. Genes with |MM| > 0.8 and |GS| > 0.5 were retained as monocyte/macrophage-associated MEblack module genes. A total of 122 genes met these criteria and were used for subsequent intersection analysis with VILI-related differentially expressed gene. The volcano plot showed the overall distribution of VILI-related differentially expressed gene (Figure 2B), and the intersection of MEblack module genes with VILI-related differentially expressed gene yielded 99 candidate genes (Figure 2C). These genes were simultaneously related to VILI status and monocyte/macrophage infiltration, suggesting that they may participate in monocyte/macrophage-mediated inflammation in VILI. KEGG analysis showed enrichment mainly in cytokine-cytokine receptor interaction, IL-17, C-type lectin receptor, TNF, and NF-κB signaling pathways (Figure 2D). GO-BP enrichment analysis highlighted biological processes related to chemotaxis, inflammatory regulation, responses to lipopolysaccharide and bacterial-derived molecules, leukocyte recruitment, myeloid cell migration, granulocyte migration, and neutrophil migration (Figure 2E).

PPI network analysis identified Ccr1-related inflammatory network features

PPI network analysis showed that several inflammatory and chemotaxis-related genes had high connectivity, including Ccr1, Il1r2, Plaur, Saa3, Ptx3, Areg, S100a8, S100a9, Cxcr2, Cxcl2, Ccl3, and Csf3r (Figure 2F). Most of these genes are associated with monocyte/macrophage chemotaxis, inflammatory factor responses, granulocyte recruitment, and tissue injury repair. The correlation heatmap of core candidate genes further showed coordinated inflammatory network features (Figure 2G). Ccr1, a chemokine receptor, was located in a core position in the network, suggesting that it may participate in the recruitment of monocyte/macrophage and other inflammatory cells into lung tissues during VILI.

Differential expression analysis based on monocyte/macrophage abundance further confirmed VILI inflammatory gene features

**Figure 3:**
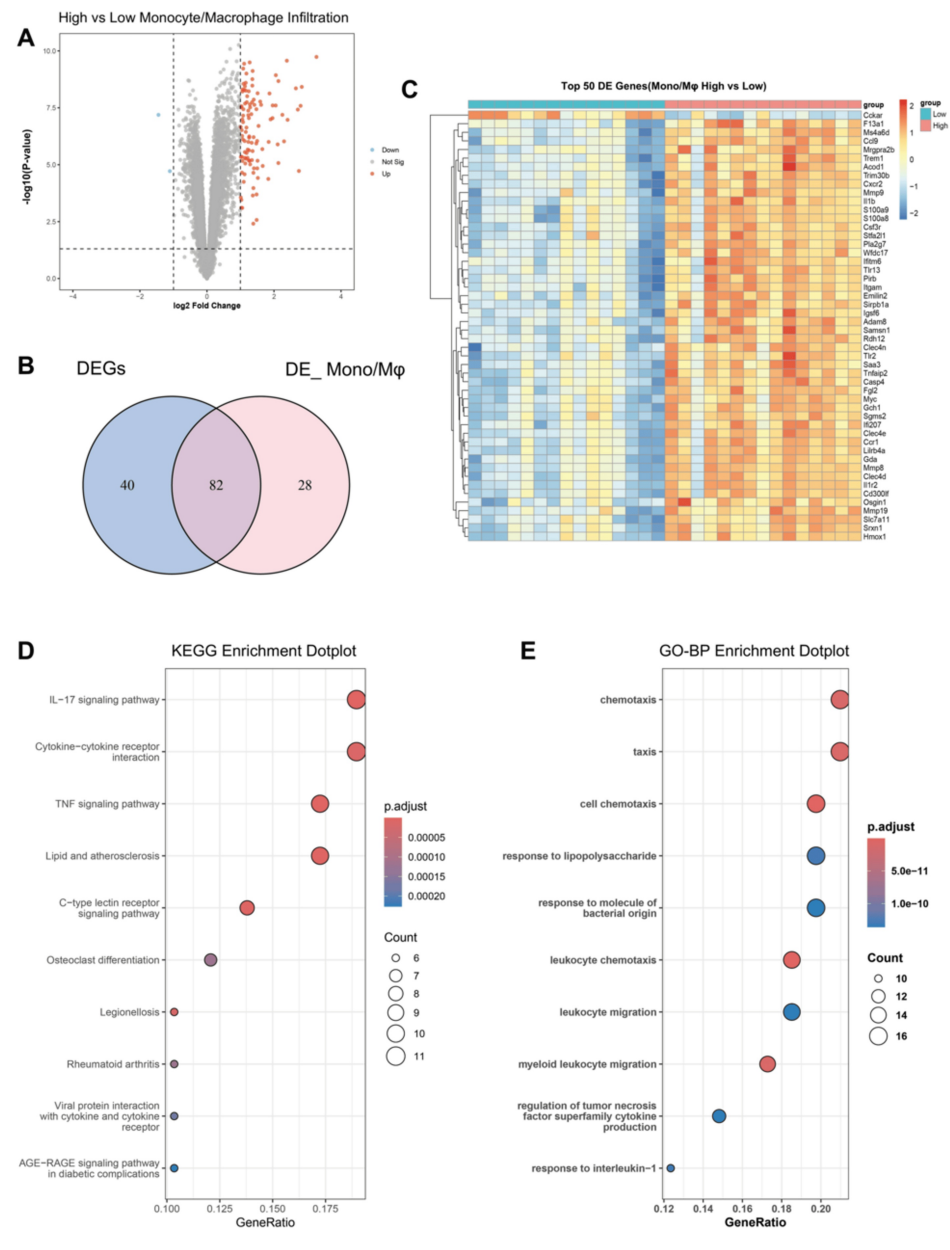
Differential genes and functional enrichment analysis based on monocyte/macrophage abundance A: Volcano plot of differentially expressed genes between high- and low-monocyte/macrophage infiltration groups. B: Intersection of VILI-related differentially expressed gene and monocyte/macrophage-related differentially expressed genes. C: Heatmap of the top 50 differentially expressed genes. D-E: KEGG and GO enrichment analyses VILI: ventilator-induced lung injury; DEGs: differentially expressed genes; Mono/Mφ: monocyte/macrophage; KEGG: Kyoto Encyclopedia of Genes and Genomes; GO: Gene Ontology; GO-BP: Gene Ontology biological process; IL-17: interleukin-17; TNF: tumor necrosis factor

To further identify genes associated with monocyte/macrophage infiltration, samples were stratified into high- and low-infiltration groups according to monocyte/macrophage abundance. Differential expression analysis showed marked gene expression differences between the two groups (Figure 3A). The intersection of monocyte/macrophage-related differentially expressed genes and VILI-related differentially expressed gene yielded 82 common genes (Figure 3B). Heatmap analysis showed that these genes were generally upregulated in samples with high monocyte/macrophage infiltration and could distinguish high- from low-infiltration states (Figure 3C). KEGG analysis revealed that these genes were mainly enriched in IL-17 signaling, cytokine-cytokine receptor interaction, TNF signaling, lipid and atherosclerosis, and C-type lectin receptor signaling pathways (Figure 3D). GO enrichment analysis highlighted biological processes associated with chemotaxis, leukocyte and myeloid cell migration, and responses to lipopolysaccharide, bacterial-derived molecules, and IL-1 (Figure 3E).

Three machine learning algorithms jointly identified Ccr1, Mmp9, and Il36g

**Figure 4:**
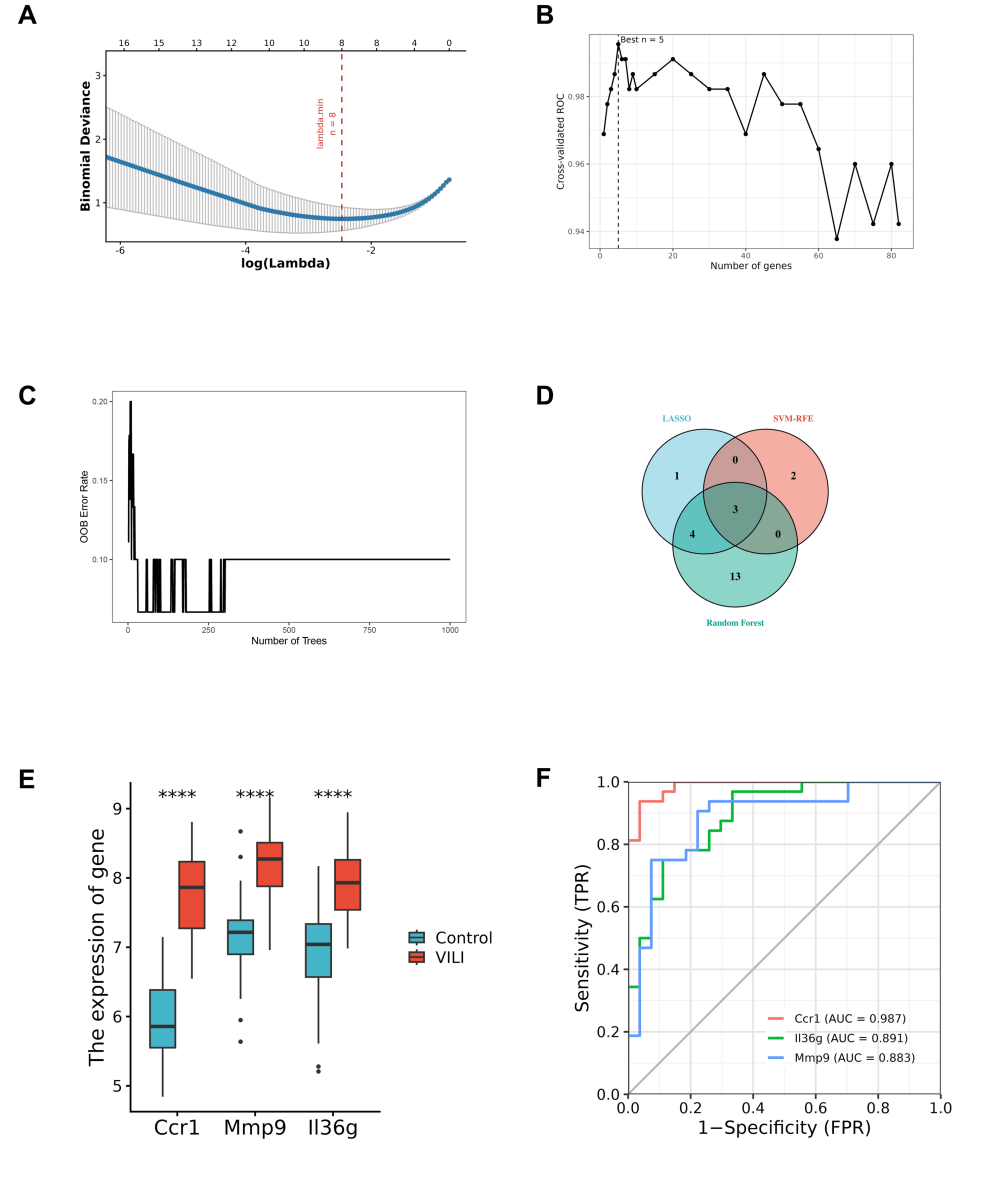
Machine learning screening of core feature genes in VILI A: LASSO regression screening of feature genes. B: SVM-RFE determination of the optimal number of feature genes. C: Random forest out-of-bag error curve. D: Intersection of the three machine-learning algorithms identifying Ccr1, Mmp9, and Il36g. E: Expression differences of core genes between VILI and control groups. F: ROC curves evaluating the discriminatory performance of core genes VILI: ventilator-induced lung injury; LASSO: least absolute shrinkage and selection operator; SVM-RFE: support vector machine-recursive feature elimination; OOB: out-of-bag; ROC: receiver operating characteristic; AUC: area under the receiver operating characteristic curve; TPR: true positive rate; FPR: false positive rate; Ccr1: C-C motif chemokine receptor 1; Mmp9: matrix metalloproteinase 9; Il36g: interleukin 36 gamma

To obtain core genes with discriminatory value, LASSO regression, SVM-RFE, and random forest algorithms were used for feature screening. LASSO regression screened candidate feature genes (Figure 4A), SVM-RFE determined the optimal feature set (Figure 4B), and random forest was used to evaluate out-of-bag error and variable importance (Figure 4C). The intersection of the three algorithms identified Ccr1, Mmp9, and Il36g as common core feature genes (Figure 4D). Expression analysis showed that Ccr1, Mmp9, and Il36g were significantly upregulated in VILI samples compared with controls, indicating that these genes were related not only to monocyte/macrophage infiltration but also to VILI status (Figure 4E). ROC curve analysis showed that all three genes had good classification ability for VILI (Figure 4F). Ccr1 showed the highest AUC of 0.987, indicating strong discriminatory performance, whereas Il36g and Mmp9 had AUC values of 0.891 and 0.883, respectively.

Experimental validation confirmed inflammatory lung injury and upregulation of core genes in VILI

**Figure 5:**
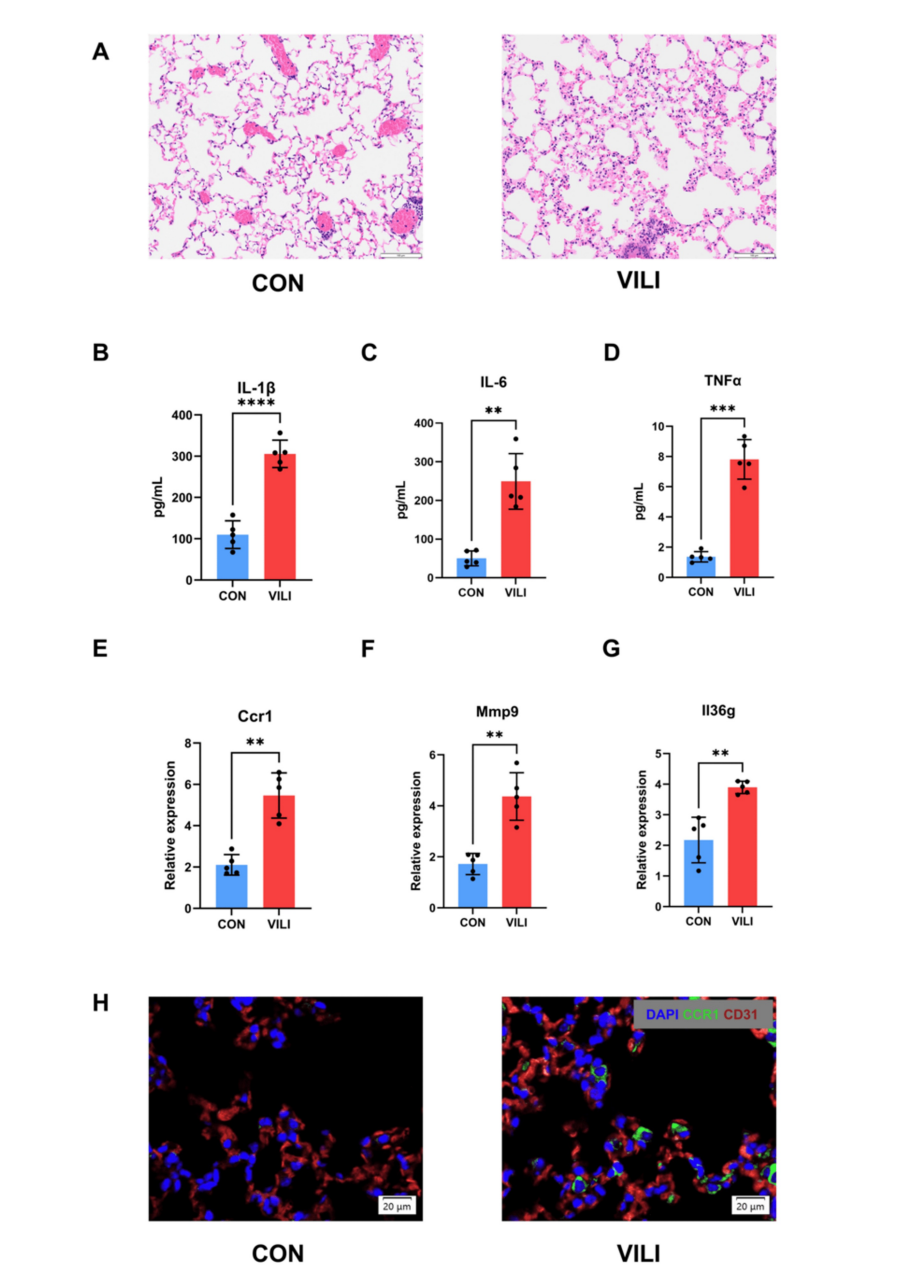
Validation of inflammatory injury and core genes in the VILI model ^**^P < 0.01. ^***^P < 0.001. ^****^P < 0.0001 A: H&E staining showing aggravated lung injury in the VILI group. B-D: Increased IL-1β, IL-6, and TNF-α levels in the VILI group. E-G: Quantitative polymerase chain reaction validation of Ccr1, Mmp9, and Il36g upregulation in VILI. H: Immunofluorescence staining showing enhanced CCR1-positive signals in the VILI group. Data are shown as mean ± SEM; n = 5 per group. P values in panels B-G were calculated using an unpaired two-tailed Student’s t-test VILI: ventilator-induced lung injury; CON: control; H&E: hematoxylin and eosin; IL-1β: interleukin-1 beta; IL-6: interleukin-6; TNF-α: tumor necrosis factor-alpha; qPCR: quantitative polymerase chain reaction; Ccr1/CCR1: C-C motif chemokine receptor 1; Mmp9: matrix metalloproteinase 9; Il36g: interleukin 36 gamma; CD31: platelet endothelial cell adhesion molecule-1; DAPI: 4′,6-diamidino-2-phenylindole; SEM: standard error of the mean

To validate the bioinformatics results, a VILI animal model was established. H&E staining showed relatively intact alveolar architecture, thin alveolar septa, and limited inflammatory cell infiltration in control lungs. In contrast, VILI lungs showed obvious inflammatory cell accumulation, disrupted alveolar structure, and local tissue injury, suggesting that mechanical ventilation induced typical inflammatory lung injury (Figure 5A). Inflammatory cytokine assays showed significantly increased IL-1β, IL-6, and TNF-α levels in the VILI group, indicating activation of a local pro-inflammatory response (Figures 5B-5D). Quantitative polymerase chain reaction further showed that Ccr1, Mmp9, and Il36g were significantly upregulated in VILI lung tissues, consistent with transcriptomic screening (Figures 5E-5G). Immunofluorescence staining showed stronger CCR1-positive signals in VILI lungs than in controls, with CCR1-positive cells distributed in alveolar septal and vascular-associated regions (Figure 5H). Combined with CD31 staining, these findings suggested that CCR1-related inflammatory signals may be associated with inflammatory cell recruitment and local barrier injury in alveolar-vascular regions.

## Discussion

This study integrated immune infiltration analysis, WGCNA, machine-learning-based feature selection, and experimental validation to identify monocyte/macrophage-related genes in VILI. The results showed marked remodeling of the pulmonary immune microenvironment, with increased monocyte/macrophage and neutrophil proportions. Consistently, the MEblack module was positively correlated with monocyte/macrophage infiltration, and the intersecting genes were enriched mainly in chemotaxis, leukocyte migration, inflammatory response regulation, and cytokine-related pathways. These findings suggest that VILI involves not only direct mechanical stress-induced tissue injury but also innate immune activation characterized by monocyte/macrophage-associated inflammatory responses.

Monocytes and macrophages play complex roles in acute lung injury. Under resolving conditions, macrophages contribute to the clearance of dead cells and tissue repair. However, excessive activation of monocytes/macrophages can promote the release of cytokines, chemokines, reactive oxygen species, and proteases, thereby recruiting neutrophils and aggravating epithelial and endothelial barrier injury [5,6]. In this study, VILI-related genes were enriched in IL-17, TNF, and NF-κB signaling pathways, supporting the involvement of innate immune inflammatory networks in VILI progression. This is also consistent with previous evidence showing that neutrophil-derived IL-17 promotes high-tidal-volume ventilation-induced lung injury [8].

Among the three identified hub genes, Ccr1 showed the strongest discriminatory performance in the integrated cohort. CCR1 is a chemokine receptor involved in inflammatory cell trafficking, including the recruitment of monocytes and macrophages. CC chemokine receptors are important regulators of inflammatory cell migration [25]. In the present study, Ccr1 was upregulated in VILI lung tissues, and CCR1-positive signals were enhanced in alveolar septal and perivascular regions. These findings suggest that CCR1 may reflect chemokine-mediated inflammatory cell accumulation during VILI. Therefore, Ccr1 may serve as a candidate marker of pulmonary inflammatory activation and a potential molecule for further investigation in monocyte/macrophage-related VILI.

Mmp9 and Il36g may represent additional inflammatory features accompanying VILI. MMP9 is associated with extracellular matrix degradation and barrier remodeling, whereas Il36g belongs to the IL-1 cytokine family and may contribute to cytokine-mediated inflammatory activation [26,27]. Together, Ccr1, Mmp9, and Il36g may reflect different but interconnected components of VILI-related inflammatory progression: Ccr1 may facilitate inflammatory cell recruitment, Mmp9 may contribute to tissue remodeling and alveolar-capillary barrier injury, and Il36g may amplify local inflammatory signaling. Their upregulation supports the presence of immune-cell accumulation, tissue remodeling, and inflammatory activation in mechanically injured lungs, although their precise cellular sources and functional roles require further study.

The enrichment of terms related to response to lipopolysaccharide and molecules of bacterial origin should not be interpreted as evidence of infection. Instead, these terms may reflect activation of innate immune pattern-recognition programs after sterile mechanical injury. Mechanical stress can trigger endogenous danger signals, leading to transcriptional responses that overlap with bacteria-associated inflammatory pathways.

From a clinical translational perspective, monocyte/macrophage-related genes and cytokine profiles may provide candidate indicators of inflammatory activation in VILI. Increased IL-1β, IL-6, and TNF-α levels may reflect complementary inflammatory processes, including inflammasome-related activation, local or systemic inflammatory responses, endothelial dysfunction, and inflammatory cell recruitment. Therefore, combining hub genes such as Ccr1, Mmp9, and Il36g with cytokine profiling may have potential value for monitoring inflammatory activity, risk stratification, follow-up assessment, and evaluation of treatment responses in mechanically ventilated patients. However, because the present findings were based on mouse transcriptomic datasets and an experimental VILI model, their diagnostic or prognostic value requires further validation in human blood, bronchoalveolar lavage fluid, or lung tissue samples.

Strengths and limitations

This study has several strengths. By combining immune infiltration analysis, WGCNA, enrichment analysis, and machine-learning methods, the screening strategy reduced reliance on differential expression analysis alone and improved the biological relevance of the candidate genes. In addition, the focus on monocyte/macrophage-related inflammation was supported by immune infiltration results, and the bioinformatics findings were further validated by histology, cytokine assays, qPCR, and CCR1 immunofluorescence staining.

This study has several limitations. First, although seven GEO datasets were integrated to increase sample size, differences in microarray platforms, ventilation settings, ventilation duration, and experimental protocols may have introduced residual heterogeneity despite batch-effect correction. Second, because the transcriptomic data were derived from whole lung tissues, the exact cellular sources of Ccr1, Mmp9, and Il36g remain unclear, and immune infiltration results should be interpreted as transcriptomic associations rather than direct cellular evidence. Third, ROC analysis was performed in the same integrated cohort used for gene discovery, without independent validation; therefore, the diagnostic performance of Ccr1 may be overestimated and should be considered exploratory. In addition, feature stability across independent cohorts could not be fully assessed. Finally, although experimental validation confirmed gene upregulation, it did not establish causality. Future studies using single-cell or spatial transcriptomics, independent validation cohorts, and CCR1-targeted interventions are needed to clarify the cellular origin and functional role of these genes in VILI.

## Conclusions

This study showed that VILI is associated with substantial remodeling of the pulmonary immune microenvironment, including increased monocyte/macrophage infiltration, neutrophil-related inflammation, and activation of chemotaxis-related inflammatory programs. Integrated bioinformatics and machine-learning analyses identified Ccr1, Mmp9, and Il36g as monocyte/macrophage-related core feature genes. Among these genes, Ccr1 showed the strongest discriminatory performance in the integrated cohort and was further supported by experimental evidence demonstrating increased CCR1-positive signals in VILI lung tissues. Overall, Ccr1 may serve as a candidate marker of monocyte/macrophage-associated inflammatory activation in VILI. Further studies using independent validation cohorts and functional experiments are needed to confirm its diagnostic value and to clarify whether CCR1-targeted interventions could modulate inflammatory cell recruitment and the progression of lung injury.

## References

[1] Beitler JR, Malhotra A, Thompson BT (2016). Ventilator-induced lung injury. Clin Chest Med.

[2] Slutsky AS, Ranieri VM (2013). Ventilator-induced lung injury. N Engl J Med.

[3] Halbertsma FJJ, Vaneker M, Scheffer GJ, van der Hoeven JG (2005). Cytokines and biotrauma in ventilator-induced lung injury: a critical review of the literature. Neth J Med.

[4] Chen L, Xia HF, Shang Y, Yao SL (2018). Molecular mechanisms of ventilator-induced lung injury. Chin Med J (Engl).

[5] Herold S, Mayer K, Lohmeyer J (2011). Acute lung injury: how macrophages orchestrate resolution of inflammation and tissue repair. Front Immunol.

[6] Lin CK, Huang TH, Yang CT, Shi CS (2021). Roles of lung-recruited monocytes and pulmonary vascular endothelial growth factor in resolving ventilator-induced lung injury. PLoS One.

[7] Huang TH, Fang PH, Li JM, Ling HY, Lin CM, Shi CS (2019). Cyclooxygenase-2 activity regulates recruitment of VEGF-secreting Ly6C(high) monocytes in ventilator-induced lung injury. Int J Mol Sci.

[8] Liao X, Zhang W, Dai H (2021). Neutrophil-derived IL-17 promotes ventilator-induced lung injury through p38 MAPK/MCP-1 pathway activation. Front Immunol.

[9] Li Z, Xiao Y, Xu L, Wang Q (2021). Mining the key genes for ventilator-induced lung injury using co-expression network analysis. Biosci Rep.

[10] Davis S, Meltzer PS (2007). GEOquery: a bridge between the Gene Expression Omnibus (GEO) and BioConductor. Bioinformatics.

[11] Leek JT, Johnson WE, Parker HS, Jaffe AE, Storey JD (2012). The sva package for removing batch effects and other unwanted variation in high-throughput experiments. Bioinformatics.

[12] Smyth GK (2005). limma: linear models for microarray data. Bioinformatics and Computational Biology Solutions Using R and Bioconductor. Bioinformatics and Computational Biology Solutions Using R and Bioconductor.

[13] Ritchie ME, Phipson B, Wu D, Hu Y, Law CW, Shi W, Smyth GK (2015). limma powers differential expression analyses for RNA-sequencing and microarray studies. Nucleic Acids Res.

[14] Newman AM, Liu CL, Green MR (2015). Robust enumeration of cell subsets from tissue expression profiles. Nat Methods.

[15] Chen Z, Huang A, Sun J, Jiang T, Qin FX, Wu A (2017). Inference of immune cell composition on the expression profiles of mouse tissue. Sci Rep.

[16] Singhania A, Graham CM, Gabryšová L (2019). Transcriptional profiling unveils type I and II interferon networks in blood and tissues across diseases. Nat Commun.

[17] Langfelder P, Horvath S (2008). WGCNA: an R package for weighted correlation network analysis. BMC Bioinformatics.

[18] Yu G, Wang LG, Han Y, He QY (2012). clusterProfiler: an R package for comparing biological themes among gene clusters. OMICS.

[19] Szklarczyk D, Gable AL, Lyon D (2019). STRING v11: protein-protein association networks with increased coverage, supporting functional discovery in genome-wide experimental datasets. Nucleic Acids Res.

[20] Friedman J, Hastie T, Tibshirani R (2010). Regularization paths for generalized linear models via coordinate descent. J Stat Softw.

[21] Breiman L (2001). Random forests. Mach Learn.

[22] Robin X, Turck N, Hainard A, Tiberti N, Lisacek F, Sanchez JC, Müller M (2011). pROC: an open-source package for R and S+ to analyze and compare ROC curves. BMC Bioinformatics.

[23] Wickham H (2016). ggplot2: Elegant Graphics for Data Analysis. New York.

[24] Livak KJ, Schmittgen TD (2001). Analysis of relative gene expression data using real-time quantitative PCR and the 2(-Delta Delta C(T)) Method. Methods.

[25] White GE, Iqbal AJ, Greaves DR (2013). CC chemokine receptors and chronic inflammation--therapeutic opportunities and pharmacological challenges. Pharmacol Rev.

[26] Davey A, McAuley DF, O'Kane CM (2011). Matrix metalloproteinases in acute lung injury: mediators of injury and drivers of repair. Eur Respir J.

[27] Peñaloza HF, van der Geest R, Ybe JA, Standiford TJ, Lee JS (2021). Interleukin-36 cytokines in infectious and non-infectious lung diseases. Front Immunol.

